# Effect of continuous theta burst stimulation on the glymphatic system, brain network and cognitive function in patients with cerebral small vessel disease

**DOI:** 10.3389/fnhum.2024.1509483

**Published:** 2025-01-21

**Authors:** Pei Dai, Hui-xian Yu, Zhao-xia Wang, Si-hao Liu, Chang-bin Liu, Guang-qing Xu

**Affiliations:** ^1^Department of Rehabilitation Medicine, Beijing Tiantan Hospital, Capital Medical University, Beijing, China; ^2^Department of Rehabilitation Medicine, Guangdong Provincial People’s Hospital, Guangdong Academy of Medical Sciences, Guangzhou, China

**Keywords:** cerebral small vessel disease, continuous theta burst stimulation, glymphatic system, brain network, cognition

## Abstract

**Objective:**

We aim to investigate the impact of continuous theta burst stimulation (cTBS) on glymphatic system (GS), brain network (BN) and cognition in cerebral small vessel disease (CSVD).

**Methods:**

This exploratory study included a small cohort of 11 patients, divided into a cTBS group (6 patients) and a sham-cTBS group (5 patients). Over a period of 2 weeks, all participants underwent cTBS to the right dorsolateral prefrontal cortex (DLPFC). The efficiency of the GS was assessed by along the perivascular space (ALPS) index. BN was measured using global efficiency (GE), characteristic path length (CPL) and clustering coefficient (*C_p_*). Cognition was evaluated by Montreal Cognitive Assessment (MoCA).

**Results:**

In the cTBS group, the ALPS index increased 4 out of 6 after treatment, compared to an increase in only 2 out of 5 in the control. Improvements in GE, CPL and *C_p_* were observed in 4 out of 6 patients in the cTBS group, whereas no improvements were noted in the control group. The MoCA scores for all patients in the cTBS improved after treatment. Additionally, completion times of the Stroop color and word test C (Stroop C) were reduced for all individuals in the cTBS group, while the control saw an increase in one case. The Digital Span Test-backward (DST-backward) scores were significantly higher in the cTBS group than those in the control.

**Conclusion:**

Applying cTBS to the DLPFC in CSVD may enhance the efficiency of brain glymphatic clearance, optimize network connectivity and improve cognitive function to a certain extent.

## 1 Introduction

Cerebral small vessel disease (CSVD) refers to a spectrum of clinical, imaging, and pathological syndromes resulting from various etiologies affecting cerebral arterioles, arterioles, capillaries, and venules (Pantoni, 2010). CSVD accounts for 25% of stroke, and its incidence increases with age (Cannistraro et al., 2019). Key cerebral imaging markers of CSVD include recent subcortical infarcts, white matter hyperintensities (WMH), lacunes, cerebral microbleeds (CMBs), enlarged perivascular spaces (EPVS), and cerebral atrophy (Wardlaw et al., 2013), which are primarily associated with vascular cognitive impairment (VCI) (Shi and Wardlaw, 2016; Lamar et al., 2022). CSVD typically impairs cognitive functions such as attention, processing speed, and executive function (EF) (Sachdev et al., 2014). Current hypotheses regarding the cognitive decline associated with CSVD suggest that the abnormal deposition of amyloid β (Aβ) and neural network damage are significant contributing factors (Kang et al., 2023; Hilal et al., 2021). Many studies have demonstrated that Aβ deposition is closely related to the occurrence of cognitive impairment (Petersen et al., 2016; Peng et al., 2016; Kamagata et al., 2022). Aβ is cleared by several mechanisms, including the cerebral glymphatic pathway (Wu J. et al., 2021; Hablitz and Nedergaard, 2021), which is a recently discovered system that plays a crucial role in removing metabolic wastes from the brain. Dysfunction in this pathway may limit the effective clearance of brain metabolic wastes such as Aβ (Rasmussen et al., 2018). As mentioned above, dysfunction of the cerebral glymphatic pathway may cause impairment of cognitive function.

Theta burst stimulation (TBS) is a specialized form of repetitive transcranial magnetic stimulation (rTMS), including continuous TBS (cTBS) and intermittent TBS (iTBS). Notably, cTBS has been shown to rapidly induce the inhibition of neural function (long-term depression, LTD) (Huang et al., 2005). In recent years, TBS has gained increased attention in improving cognitive dysfunction in post-stroke patients (Tsai et al., 2020; Li et al., 2022). The dorsolateral prefrontal cortex (DLPFC) is critical in managing various behavioral tasks and is essential for several cognitive processes, including attention (Stonsaovapak et al., 2020), working memory (Vékony et al., 2018), decision making (Obeso et al., 2021), and inhibitory control (McNeill et al., 2018; Chen et al., 2021). Damage to circuits involving the DLPFC can impair EF (Osada et al., 2019). Therefore, targeting the DLPFC through interventions like rTMS and TBS could potentially modulate cognitive control. The neural effects of rTMS depend on the frequency of stimulation. High frequency stimulation over the left DLPFC is the classic stimulation mode of rTMS for cognitive impairment. However, TBS are thought to be the more “naturalistic” pattern of stimulation and applied over a fraction of the time than rTMS. Compared with the excitatory effect of iTBS, cTBS may affect cognitive function through different mechanisms. In previous studies, the effectiveness of unilateral cTBS regimens on right DLPFC in improving cognitive function or depression remains controversial (Langenbach et al., 2019; Maier et al., 2018). Some studies even suggest that cTBS may reduce or inhibit cognitive control when applied to the right DLPFC (Maier et al., 2018; McNeill et al., 2018). While other research indicates that cTBS could have a subtle yet discernible negative effect on EF task performance, and these effects being pronounced when stimulating the left DLPFC rather than the right (Wu X. et al., 2021). A study by Langenbach et al. (2019) involving 100 healthy college students, confirmed that cTBS treatment on the right DLPFC did not change the subjects’ continuous decision-making processes. In animal models, however, cTBS can improve the spatial cognitive function in mice, which may be attributed to the regulation of aquaporin-4 polarization, increased local cerebral blood perfusion, reduced inflammatory response, and a significant decrease in infarction volume, thereby improving the function of the cerebral glymphatic system and cognitive function (Wu et al., 2022; Vékony et al., 2018). Additionally, recent studies have found that cTBS can improve the learning and memory performance of stroke mice by activating GABA neurons, crucial neurotransmitters for brain reorganization and repair, and increasing the frequency of micro-excitatory postsynaptic currents (Feng et al., 2020). These findings underscore the cTBS’ potential of effectively regulating the glymphatic pathway to clear metabolic waste, mitigate Aβ deposition, prevent neural network damage, and enhance cognitive function in patients with CSVD-related cognitive impairment.

In this study, we explored the effects and possible mechanisms of cTBS on the improvement of cognitive function in CSVD patients. We evaluated the glymphatic system and measured several indexes, including GE, CPL, and *C_p_*, to assess the global and local brain network function and efficacy. This research contributes to understanding cTBS’s therapeutic potential and provides valuable insights for clinical intervention strategies.

## 2 Materials and methods

### 2.1 Research design

Due to the limited number of patients enrolled (six in the cTBS group and five in the sham-cTBS group), this study was conducted as an exploratory investigation. Consequently, only descriptive analysis was performed, rather than statistical analysis, to evaluate the outcomes.

### 2.2 Patient selection

A total of 11 CSVD patients aged between 40 and 80 years were enrolled in our study. These patients were hospitalized in the Rehabilitation Department from January 2021 to April 2023. The cohort comprised 8 males and 3 females, and all participants signed informed consent. This study was approved by the Ethics Committee of Beijing Tiantan Hospital. Upon admission, all subjects underwent a routine head magnetic resonance imaging (MRI) examination. The magnetic resonance model is the German Siemens 3.0 T Trio magnetic resonance imaging system and the scan sequences included T1-weighted images, T2-weighted images, diffusion weighted imaging (DWI), fluid attenuated inversion recovery (FLAIR), and susceptibility weighted imaging (SWI).

Inclusion criteria: (1) diagnosis consistent with the 2010 definition of CSVD (Pantoni, 2010), with imaging manifestations including lacunae, new subcortical infarcts, WMH, EPVS, cerebral microbleeds, and cerebral atrophy (Wardlaw et al., 2013); (2) patients meeting the diagnostic criteria of “Chinese guidelines for diagnosis and treatment of cognitive dysfunction related to cerebral small vessel disease (2019)” (Peng, 2019); (3) education level of 6 years or more; (4) age between 40 and 80 years; (5) voluntary signing of informed consent by patients or their family members.

Exclusion criteria: (1) diagnosed with large artery atherosclerosis stroke; (2) inability to cooperate with cognitive assessment; (3) presence of other conditions that may affect cognitive function; and (4) inability to undergo head MRI examination for various reasons.

The study was conducted following the Declaration of Helsinki.

### 2.3 Study process

Initially, 12 patients with CSVD-related cognitive impairment were divided into two groups in a 1:1 ratio: the cTBS group and the sham-cTBS group, with six patients in each. In the sham-cTBS group, one patient was excluded early due to being discharged. All remaining patients received complete treatment and assessment of cognitive scale and head MRI before and after treatment.

### 2.4 Outcome

#### 2.4.1 Primary outcome

1. ALPS index: The calculation formula is as follows:

$$\mathrm{ALPS index}=\mathrm{mean}\;\left(\mathrm{Dxproj,Dxassoc}\right)/\mathrm{mean}\;\left(\mathrm{Dyproj,Dzassoc}\right)\text{.}$$

A higher ratio indicates more diffusion of water molecules in the direction of the paravascular space, suggesting improved clearance efficiency of the cerebral lymphatic pathway.

1. GE: A larger GE value indicates higher efficiency within the brain network.
2. MoCA scale (Nasreddine et al., 2005): this scale covers visuospatial and executive function, naming, memory, attention, language, abstraction, delayed recall, and orientation, with a total possible score of 30 points. Scores below 26 points indicate cognitive impairment. To account for the influence of education level on the assessment results, an additional point is added for subjects with less than 12 years of education. Higher MoCA scores reflect better cognitive function in patients.

#### 2.4.2 Secondary outcome

1. CPL: A shorter CPL indicates higher efficiency.
2. *C_p_*: Larger *C_p_* indicates closer local connectivity within the brain network.
3. SCWT: Records the time required for all patients to complete the three SCWT cards. Shorter completion times indicate better inhibitory control of executive functions.
4. Trail Making Test (TMT): Records the time required for all patients to complete parts A and B of the task. Shorter test times suggest improved set-shifting ability.
5. DST: This test includes digital forward and digital backward. Higher scores reflect better memory refreshment, attention, and working memory capabilities.

### 2.5 TMS procedure

The resting motor threshold (RMT) was measured before treatment. The motor evoked potentials (MEPs) from the first dorsal interosseous muscle were recorded using electromyography electrodes. The RMT is defined as the minimum stimulus intensity required to elicit a target MEP in at least 5 out of 10 trials, with each MEP having an amplitude exceeding 50 μV.

#### 2.5.1 cTBS group

Patients in this group received routine treatment along with cTBS. The treatment utilized the Yiruide CCY-IA magnetic stimulation device (Wuhan Yiruide Medical Equipment New Technology Co., Ltd., China). Each patient was seated comfortably and instructed to keep his head as still as possible after full communication. The researcher held an “8” shaped coil and placed it on the right DLPFC of the patient, ensuring the center of the coil was tangent to the skull. The cTBS mode was adopted with the following specific parameters: the session consisted of three-pulse bursts at 50 Hz repeated at 5 Hz, the stimulation duration was 40s, the total stimulation number was 600 pulses, and intensity was set at 80% of the MT. Treatments were administered once a day, 5 days a week, for a total duration of 2 weeks. The training can be conducted after or before the application of cTBS.

#### 2.5.2 Sham-cTBS group

Patients in this group received routine treatment and sham-cTBS. For the sham-cTBS procedure, the researcher placed the “8” shaped coil on the right DLPFC of the patient, and the coil was perpendicular to the skull. All stimulation parameters and treatment times were exactly the same as those used in the cTBS group.

#### 2.5.3 Routine cognitive training

According to the assessment results of each patient’s cognitive function, therapists developed a targeted cognitive training program that includes one or more of the following areas: attention training, executive function training, memory training, visuospatial function training, etc. Each training session lasted approximately 30 min and was conducted twice a day, 5 days a week, for a total duration of 2 weeks.

### 2.6 Statistical analysis

Given the small sample size of this study, traditional statistical methods were not applicable for effective analysis. Consequently, only descriptive analysis was conducted to compare the outcomes before and after treatment in both the cTBS and sham-cTBS groups.

## 3 Results

### 3.1 General information

The study enrolled a total of 11 patients. In the cTBS group, there were 6 patients aged between 63 and 75 years, with educational backgrounds ranging from 6 to 16 years; this group comprised 4 males and 2 females. The sham-cTBS group included 5 patients aged between 58 and 75 years, also with 6 to 16 years of education, consisting of 4 males and 1 female.

### 3.2 Primary outcome

#### 3.2.1 Changes in ALPS index in both groups before and after treatment

In the cTBS group, the ALPS index increased in of 4 out of 6 patients after treatment compared to their baseline measurements. In contrast, in the sham-cTBS group, only 2 out of 5 patients showed an increase in the ALPS index following treatment (Table 1).

**Table 1:** Comparison of ALPS index before and after treatment in 11 patients.

Group		ALPS index		
		Before treatment	After treatment	Change from the baseline
cTBS	Patient 1	0.5149	0.7440	0.2291^a^
	Patient 2	0.4010	0.7601	0.3591^a^
	Patient 3	1.3401	0.8201	−0.5200
	Patient 4	0.8656	1.1927	0.3271^a^
	Patient 5	1.7784	2.1569	0.3785^a^
	Patient 6	1.4395	0.9268	−0.5127
Sham-cTBS	Patient 1	1.9923	1.9487	−0.0436
	Patient 2	3.8549	0.7207	−3.1342
	Patient 3	0.3528	0.7639	0.4111^a^
	Patient 4	0.6836	0.5554	−0.1282
	Patient 5	0.4684	1.5107	1.0423^a^

ALPS, along the perivascular space.

a Indicates that the ALPS index increased after treatment compared with that before treatment.

#### 3.2.2 Changes in GE of the two groups before and after treatment

In the cTBS group, 4 out of 6 patients exhibited an increase in GE post-treatment, indicating improved efficiency within the brain network. Conversely, all 5 patients in the sham-cTBS group experienced a decrease in GE to varying degrees after treatment (Table 2).

**Table 2:** Comparison of GE before and after treatment in 11 patients.

Group		GE		
		Before treatment	After treatment	Change from the baseline
cTBS	Patient 1	0.767	0.722	−0.045
	Patient 2	0.767	0.753	−0.014
	Patient 3	0.788	0.803	0.015^a^
	Patient 4	0.763	0.778	0.015^a^
	Patient 5	0.737	0.746	0.009^a^
	Patient 6	0.787	0.801	0.014^a^
Sham-cTBS	Patient 1	0.795	0.792	−0.003
	Patient 2	0.787	0.503	−0.284
	Patient 3	0.795	0.779	−0.016
	Patient 4	0.794	0.772	−0.022
	Patient 5	0.793	0.785	−0.008

GE, global efficiency.

a Indicates that the GE increased after treatment compared with that before treatment.

#### 3.2.3 Changes in CPL of the two groups before and after treatment

In the cTBS group, 4 out of 6 patients demonstrated a decrease in CPL following treatment, suggesting enhanced transmission speed and efficiency within the brain network. On the other hand, the CPL increased in all 5 patients of the sham-cTBS group compared to before treatment, indicating a reduction in network efficiency (Table 3).

**Table 3:** Comparison of CPL before and after treatment in 11 patients.

Group		CPL		
		Before treatment	After treatment	Change from the baseline
cTBS	Patient 1	1.458	1.563	0.105
	Patient 2	1.458	1.488	0.030
	Patient 3	1.422	1.389	−0.033^a^
	Patient 4	1.468	1.438	−0.030^a^
	Patient 5	1.525	1.501	−0.024^a^
	Patient 6	1.417	1.390	−0.027^a^
Sham-cTBS	Patient 1	1.400	1.407	0.007
	Patient 2	1.421	2.320	0.899
	Patient 3	1.406	1.437	0.031
	Patient 4	1.406	1.454	0.048
	Patient 5	1.404	1.421	0.017

CPL, characteristic path length.

a Indicates that the CPL decreased after treatment compared with that before treatment.

#### 3.2.4 Changes in *C_p_* of the two groups before and after treatment

In the cTBS group, 4 out of 6 patients showed an increase in *C_p_* after treatment, indicating enhanced local connectivity within the brain network. In contrast, all 5 patients in the sham-cTBS group experienced decreases in *C_p_* of different degrees after treatment (Table 4).

**Table 4:** Comparison of *C_p_* before and after treatment in 11 patients.

Group		*C_p_*		
		Before treatment	After treatment	Change from the baseline
cTBS	Patient 1	0.748	0.746	−0.002
	Patient 2	0.758	0.733	−0.025
	Patient 3	0.782	0.809	0.027^a^
	Patient 4	0.760	0.779	0.019^a^
	Patient 5	0.714	0.718	0.004^a^
	Patient 6	0.770	0.787	0.017^a^
Sham-cTBS	Patient 1	0.774	0.769	−0.005
	Patient 2	0.777	0.566	−0.211
	Patient 3	0.793	0.777	−0.016
	Patient 4	0.778	0.776	−0.002
	Patient 5	0.780	1.759	−0.021

*Cp, clustering coefficient.*

a Indicates that the *C_p_* increased after treatment compared with that before treatment.

### 3.3 Secondary outcome

MoCA score, Stroop C time, DST score, TMT time: Table 5 shows the cognitive function data for the cTBS and sham-cTBS groups before and after treatment. Table 6 outlines the changes from baseline in cognitive function for both groups before and after treatment. As shown in Tables 5, 6, the MoCA scores for all 6 patients in the cTBS group improved to various extents compared to before treatment, with the average change from baseline being slightly higher than that in the sham-cTBS group. The completion times for the Stroop C test in the cTBS group decreased for all 6 patients with average changes from baseline greater than those in the sham-cTBS group.

**Table 5:** The cognitive function of the two groups before and after treatment.

Group		MoCA (score)		Stroop C (s)		DST-forward		DST-backward		TMT-A (s)		TMT-B (s)	
		B	A	B	A	B	A	B	A	B	A	B	A
cTBS	Patient 1	14	17	231	167	8	8	2	4	77	57	252	205
	Patient 2	22	24	141	84	9	9	4	5	84	71	262	192
	Patient 3	25	27	105	79	8	8	3	5	60	51	150	130
	Patient 4	13	15	189	163	9	9	3	3	118	95	619	485
	Patient 5	24	26	131	83	9	9	3	4	75	88	252	232
	Patient 6	20	23	87	73	6	6	3	3	80	81	262	241
Sham-cTBS	Patient 1	15	15	155	143	8	8	4	5	67	94	252	294
	Patient 2	10	18	94	109	6	7	3	3	167	115	525	496
	Patient 3	25	27	79	68	9	9	5	5	63	82	150	150
	Patient 4	24	25	122	104	8	8	6	6	73	78	180	201
	Patient 5	22	23	121	118	8	8	4	5	92	93	219	210

MoCA, Montreal Cognitive Assessment; DST, Digital Span Test; TMT, Trail Making Test; B, before treatment; A, after treatment.

**Table 6:** The changes from baseline of cognitive function of the two groups before and after treatment.

Group		MoCA (score)	Stroop C (s)	DST-forward	DST-backward	TMT-A (s)	TMT-B (s)
		Change from baseline	Change from baseline	Change from baseline	Change from baseline	Change from baseline	Change from baseline
cTBS	Patient 1	3	−64	0	2	−20	−47
	Patient 2	2	−57	0	1	−13	−70
	Patient 3	2	−26	0	2	−9	−20
	Patient 4	2	−26	0	0	−23	134
	Patient 5	2	−48	0	1	13	−20
	Patient 6	3	−14	0	0	1	−21
Sham-cTBS	Patient 1	0	−12	0	1	27	42
	Patient 2	8	15	1	0	−52	−29
	Patient 3	2	−11	0	0	19	0
	Patient 4	1	−18	0	0	5	21
	Patient 5	2	−3	0	1	1	−9

MoCA, Montreal Cognitive Assessment; DST, Digital Span Test; TMT, Trail Making Test.

However, the completion time for the Stroop C test increased for one patient in the sham-cTBS group compared to before treatment. The DST-backward scores and the average changes from baseline in TMT times for the cTBS group were significantly higher than those in the sham-cTBS group. Specifically, the completion times for TMT-A and B in the sham-cTBS group increased after treatment compared to before.

## 4 Discussion

The findings from this study revealed significant differences between the cTBS group and the sham-cTBS group across various metrics. In the cTBS group, the ALPS index increased for 4 out of 6 patients after treatment, while only 2 out of 5 patients in the sham-cTBS group showed an increase. Similarly, GE improved in 4 out of 6 patients in the cTBS group, contrasting with a decline in all 5 patients in the sham-cTBS group. CPL decreased in 4 out of 6 patients in the cTBS group, whereas it increased to different degrees in all 5 patients in the sham-cTBS group. The *C_p_* also increased in 4 out of 6 cTBS patients, indicating enhanced local connectivity, while it decreased to different levels in all patients of the sham-cTBS group. The MoCA scores improved to various degrees for all patients in the cTBS group, with an average change from baseline slightly superior to that of the sham group. Completion times for the Stroop C test decreased to various levels for all cTBS patients, and the average changes from baseline were higher than that in the sham-cTBS group. However, one patient in the sham-cTBS group showed increased completion time following treatment. DST-backward scores and average changes from baseline in TMT time for the 6 patients in the cTBS group were significantly higher than those in the sham-cTBS group. Specifically, the complete times for TMT-A and B in the sham-cTBS group increased after treatment compared to before treatment.

The glymphatic system primarily relies on aquaporin 4 (AQP4) located on the astrocyte end-foot for solute transport (Iliff et al., 2015; Mestre et al., 2020). Growing evidence has demonstrated that multiple imaging markers of CSVD disrupt the function of the cerebral glymphatic system, reducing its efficiency in cleaning metabolic wastes. One significant pathway for clearing Aβ and other metabolic waste in CSVD is the glymphatic pathway. Therefore, dysfunction of this system can decrease the scavenging ability of toxic metabolic waste in the interstitial fluid, leading to tissue hypoxia, damage, and clinical manifestations such as cognitive function impairment (Yamada et al., 2019; Kang et al., 2023). This impairment may also stem from the accumulation of various metabolic wastes, vascular endothelial dysfunction, and the destruction of the blood-brain barrier (BBB) (Jiang et al., 2021). The abnormal deposition of Aβ may be observed in all patients with CSVD-related cognitive impairment and is closely associated with the development of such cognitive deficits. Although animal studies have confirmed that cTBS can enhance the spatial cognitive functions in mice by increasing local cerebral blood perfusion, reducing the inflammatory response, and significantly decreasing infarction volume, which in turn regulates the polarization of hydroacorin-4 and improves the function of the cerebral glymphatic system (Wu et al., 2022; Vékony et al., 2018), there was no literature reporting the effects of cTBS improving the function of the cerebral glymphatic system in patients. Our study’s findings indicated that a higher proportion of patients in the cTBS group showed improvement in the ALPS index compared to the sham-cTBS group. This aligns with previous animal studies and suggests a potential positive effect of cTBS on the brain glymphatic system.

Network efficiency, measuring the information transfer efficiency of the brain network, plays an important role in explaining cognitive decline in CSVD. Tuladhar et al. (2020) conducted a prospective single-center cohort study involving 277 non-dementia patients with CSVD and found a significant correlation between the decline in GE in the severe WMH group and a decrease in psychomotor velocity, suggesting that baseline GE is associated with all-cause mortality. This relationship may reflect the overall health of individuals with CSVD. Furthermore, other studies have observed (Frey et al., 2021) that as the CSVD load increases, GE decreases and *C_p_* increases, leading to the weakness in the small-world topology. Therefore, these changes suggest a reduced capacity for integrating distributed information when the CSVD load increases. In our study, among the 6 patients in the cTBS group, GE increased in 4 patients after treatment, CPL decreased in 4 patients, and *C_p_* increased in 4 patients. Conversely, in the sham-cTBS group, GE and *C_p_* decreased in all 5 patients, CPL increased after treatment. We speculated that without additional intervention, the GE in CSVD patients might progressively decline, CPL might increase, and *C_p_* might decrease. While after active intervention, the GE, CPL, and *C_p_* in CSVD patients might be improved correspondingly, which thus leads to faster information transmission across the brain network and increased local connectivity.

Based on our results, we hypothesize that the cTBS applied to the right DLPFC might improve the overall cognitive, executive, or attention functions in CSVD patients. However, due to the lack of systematic and robust statistical analysis, the statistically significant effects of cTBS on cognitive functions remain uncertain. At the same time, the recovery of cognitive function tends to be slower than that of motor function, indicating that the benefits of short-term intervention on cognitive functions may be limited. Prolonged treatment time and increased follow-up frequency could provide a more comprehensive evaluation of the long-term effects of cTBS treatment.

The index of brain network function is potentially linked to cognitive function and may influence it substantially. Li et al. (2021) recruited 32 patients with mild cognitive impairment (MCI) and 28 patients with normal cognition and evaluated their topological features of the structural connection network. The analysis revealed that MCI patients had significantly lower *C_p_* in certain brain regions, like the left middle frontal gyrus, right superior parietal gyrus, and right inferior parietal gyrus, compared to control. Additionally, these patients exhibited a notably shortened path length in the left paracentral lobule Shen et al. (2020) assessed 19,346 individuals with normal neurological function using MRI diffusion imaging and cognitive tests. Their mediated effect analysis indicated that GE had a significant indirect impact on cognition through vascular load, suggesting that GE played a partial mediating role between vascular load and cognition. Furthermore, research on children with attention-deficit/hyperactivity disorder (ADHD) showed that longer CPLs negatively impact visuospatial working memory performance in both normally developing children and those with ADHD (Arrington et al., 2022). Given that abnormal deposition of Aβ and the damage of neural network structure and function are common pathological mechanisms in cognitive impairment (Kang et al., 2023; Hilal et al., 2021), improving the ALPS index may correlate with cognitive function enhancement. The latest study conducted by Hsu et al. (2023) also found that the ALPS index played an important mediating role between regional standardized intake ratios of Aβ and tau images and cognitive dysfunction, even after adjusting for multiple covariates in AD-related brain regions. Based on these findings, we believe that cTBS could improve cognitive function in patients with CSVD-related cognitive impairment by improving the ALPS index and both global and local efficiency of the brain network.

This study has several limitations. First, the sample size was small. Due to the limited number of participants, routine statistical analysis could not be conducted, and the improvements in cerebral glymphatic clearance efficiency and global brain efficiency in CSVD patients were primarily inferred from response rates before and after treatment. This constraint diminishes the validity and persuasiveness of our results. To enhance the reliability and generalizability of our findings, a key objective for our next study is to increase the sample size. This expansion will allow us to conduct comprehensive statistical analyses and confirm the preliminary results observed in the current research. Second, given that CSVD is a whole-brain disease, the use of a figure-eight coil targeting the right DLPFC in cTBS mode may not effectively address the global nature of this condition. Future research will explore the use of circular coils, which may provide more comprehensive treatment outcomes for CSVD patients. Third, in this study, GE, CPL, and *C_p_* were used to assess brain network function, along with fractional anisotropy (FA) and mean diffusivity (MD). To enhance the analysis of therapeutic effects, we plan to integrate ROI-based functional connectivity analysis from resting-state functional magnetic resonance imaging (fMRI) into our evaluations. This approach will allow for a more comprehensive assessment of network connectivity. Fourth, the treatment duration in this study was relatively short, with cTBS administered once daily for 2 weeks. While previous studies have shown significant improvements in executive functions with 2 weeks of TBS treatment (Cheng et al., 2016). Longer durations of TMS stimulation (e.g., over 4 weeks) have been suggested in other studies. Future protocols will consider extending the treatment period beyond 2 weeks to potentially achieve more substantial cognitive improvements. Fifth, the evaluation of cognitive function in this study lacked a follow-up phase, with the entire treatment cycle lasting only 2 weeks without subsequent assessments. Future studies will include extended treatment durations and regular follow-up sessions to evaluate the treatment effects objectively.

In summary, our existing results suggest that cTBS targeting the right DLPFC in patients with CSVD-related cognitive impairment may enhance glymphatic clearance efficiency by increasing the ALPS index. Additionally, cTBS appears to improve brain network efficiency through increased GE, reduced CPL, and enhanced *C_p_*, potentially improving cognitive function. To build on these preliminary results, future research will focus on increasing the sample size, utilizing different stimulation coils, expanding the evaluation metrics, and implementing regular patient follow-ups.

## Data Availability

The original contributions presented in the study are included in the article/supplementary material, further inquiries can be directed to the corresponding author.
